# A genetic tool to manipulate litter size

**DOI:** 10.1186/1742-9994-11-18

**Published:** 2014-02-24

**Authors:** Manuela Ferrari, Anna K Lindholm, Barbara König

**Affiliations:** 1 Institute of Evolutionary Biology and Environmental Studies, University of Zurich, Winterthurerstrasse 190, 8057 Zurich, Switzerland

**Keywords:** Litter size manipulation, House mouse, *t* haplotype, Optimal litter size

## Abstract

**Introduction:**

Experimental litter size manipulations are often not problem free. Typically conducted shortly after birth or oviposition, they do not account for the energy already invested into the production of the offspring. Such effects make it difficult to interpret the results from experimental litter size manipulations and therefore to study optimality of litter or clutch size, a long debated topic in evolutionary biology.

**Results:**

We propose the use of a mating design based on a selfish genetic element, the *t* haplotype, to reduce litter size in an eutherian mammal, the house mouse. Most *t* haplotypes are recessive lethal and therefore lead to the death of all homozygous embryos. Litter sizes can be reduced by up to 50% by pairing a +/*t* female with a +/*t* male instead of a +/+ male.

**Conclusions:**

This method allows litter size manipulation before birth without the use of invasive techniques, therefore providing an excellent tool for studying optimal litter size and ultimately helping to understand life history strategies.

## Introduction

Reproduction is a key feature of life and ultimately determines the success of an individual. At any point in time an animal should therefore optimise its reproductive effort to maximise lifetime reproductive success. Several trade-offs play an important role in this process and determine to a large extent the life history of an animal. Pianka [1] described the most important trade-offs with three simple, but crucial questions: when should an individual reproduce, how much should it invest into the current reproductive event and how much into one single offspring? The number of offspring produced by birds and mammals per reproductive event has been widely investigated over the last decades. Optimal litter or clutch size nevertheless remains puzzling as it is likely to be determined by the current environment, the trade-off between current and future reproductive efforts, as well as by the trade-off between the number and the quality of the offspring [2].

### Testing optimality of litter or clutch size

Already in the first half of the 20th century theories were developed to explain the huge variation observed in clutch size in birds. Lack [3] proposed that survival probability decreases with increasing clutch or litter size, because the amount of food parents can provision to their offspring is limited. The “Lack clutch” is therefore defined as the clutch size which fledges the largest number of offspring. In the following years Lack’s theory has been refined and the above mentioned trade-offs have been incorporated [4,5]. The most common approach to test the assumptions of the “Lack clutch” or to investigate optimality of clutch or litter size is to manipulate the number of offspring to assess whether this reduces or increases the reproductive success of the parents and the offspring. A variety of manipulative experiments have been conducted in birds, with contrasting results. For example, Styrsky *et al.*[6] found that brood size enlargement in spotted antbirds (*Hylophylax naevioides*) increases juvenile mortality after fledging, whereas brood size reductions resulted in the opposite effect. Other studies found a delay of egg-laying and a decrease in the number of successfully reared young in the next brood for rooks (*Corvus frugilegus L.*) with experimentally enlarged broods [7]. A meta-analysis on 42 brood size manipulation experiments, on the other hand, found no evidence for the Lack hypotheses. Brood size enlargement did not lead to a reduction in the number of fledglings [8]. Optimal litter size theory has also been applied to other vertebrates (mammals [9]; reptiles [10]) and invertebrates [11].

In mammals, litter size manipulations are usually conducted shortly after birth by adding or removing pups of similar age. Such manipulations affected the growth rate of offspring in rodents (white-footed mice (*Peromyscus leucopus*) [12], wild bank voles (*Myodes glareolus*) [13,14]) and the future reproductive success of females and their daughters (house mice (*Mus musculus domestics*) [15]). Other studies, in contrast, did not observe an effect of litter size manipulation on offspring condition (ground squirrels (*Spermophilus columbianus*) [16]) or female future reproduction (wild bank voles [13,14], ground squirrels [16]). Correlational data also suggests that there is no such trade-off (northern grasshopper mice (*Onychomys leucogaster*[17]). This discrepancy between different studies and methods (observational, versus experimental litter size manipulations) may indicate that postpartum manipulation of offspring number does not reflect a “naturally” large or small litter size. If females give birth to a litter size that is optimised to their current physiology and condition, manipulation of number of pups directly after birth will not result in standardization of lactational burden for different females (for a review see [18], and next section).

### Problems associated with experimental litter size manipulations

One main problem of clutch or litter size manipulation experiments is that they do not account for the energy already invested into the production of the offspring. The cost of egg production and incubation in birds was largely ignored, until Monaghan *et al.*[19] showed that it can have a substantial effect and should not be overlooked. In altricial house mice, energy demand increases during gestation by 49.2% (compared to nonreproducing females [20]). Such an increase is substantial, although lactation comes at even higher costs (house mice [20], bank voles [21]).

Pregnancy in eutherian mammals further differs from the pre-incubating phase in egg-laying birds by its effect on the mother’s hormones and behaviour. Mammary development begins already during gestation, and in utero litter size directly affects hormone levels (goats [22-24], mice [25]), mammary gland size (sheep [26], goats [22], mice [27]) and therefore likely also milk yield after birth (goats [23]). In addition, body weight of pregnant females increases with increasing prepartum litter size or litter mass [28]. Such an effect may have consequences for later lactation since heavier females produce more milk than smaller ones [29].

Despite the influence of in utero number of pups on maternal physiology and behaviour, adjustment to modified postpartum litter size is possible. Experimental litter size manipulation after birth revealed compensatory mammary growth in the first days of lactation, suggesting an ability to adjust milk production to changing litter sizes after birth [27-29]. Nevertheless, to what extent pre- and postpartum litter sizes influence maternal behaviour and lactation remains controversial. Analysing that question requires methods to manipulate litter size during gestation. One option is to surgically remove embryos at an early stage of the pregnancy (house mice [30]). This surgical method, however, is very invasive and the effects of the surgery difficult to control. Similar problems could arise after the removal of one of the ovaries prior to breeding. This method has been used in pigs to reduce litter size [31].

### The *t* haplotype as a tool to manipulate litter size

As an alternative, we propose here to use the *t* haplotype as a genetic tool to reduce litter size in an eutherian mammal, the house mouse, which is widely used as a laboratory animal. This method allows for a predictable noninvasive litter size reduction without postnatal interference. The *t* haplotype is a selfish genetic element occurring in natural house mouse populations (for a review see [32]). It is located on chromosome 17 and consists of four linked inversions, spanning approximately one third of the whole chromosome [32]. The *t* haplotype has been described for all four subspecies of the house mouse (*Mus m. domesticus, Mus m. musculus, Mus m. castaneus and Mus m. bactrianus*) [32,33]. Gene products of the *t* haplotype affect the development of the flagella of wild type sperm during spermatogenesis in +/*t* males, leading to a transmission ratio distortion with a *t* gamete transmission of up to 99% to the offspring [32]. In females *t* gamete transmission follows the classical Mendelian rule with on average 50% of the gametes receiving the *t* haplotype. By amplifying and scoring a genetic marker (*Hba-ps4*) associated with the *t* haplotype [33], this selfish genetic element can easily be identified. The *t* can be found in many wild populations and several different *t* variants are commercially available (to give an example: mouse strains *t*^*w*5^ (RBRC01202) and *t*^*w*5*G*^ (RBRC01203) from the Experimental Animal Division of the RIKEN BioResource Center). Because of the transmission ratio distortion in males, the *t* can be crossed into a population or specific strain within a rather short time (see [34]).

Most of the different *t* variants carry recessive lethals, causing the death of homozygous individuals in utero. The stage in which lethality occurs varies between *t* variants, but most often it happens around day 9 or 10 of pregnancy [35]. At this stage embryos are typically between 1.2 mm [day 9] and 3.9 mm [day 10] in size [36]. Nagasawa *et al.*[30] surgically adjusted the number of foetuses at day 8 of pregnancy in mice. They sacrificed the females at day 19 of pregnancy and analysed mammary development. The indices used to measure mammary development correlated positively with the number of embryos left after surgery suggesting that prepartum litter size (after day 8 of pregnancy) quantitatively influenced the development of the mammary gland tissue. The litter size reduction due to the recessive lethal nature of the *t* haplotype, acting in the first half of the gestation period as described above, should therefore still allow for adjustment of prenatal mammogenesis to the number of surviving embryos. It is exactly the recessive lethal property of the *t* haplotype that can be used as an instrument to reduce litter size, without interfering after birth, or applying invasive surgery to remove foetuses.

## Results and discussion

**Figure 1:**
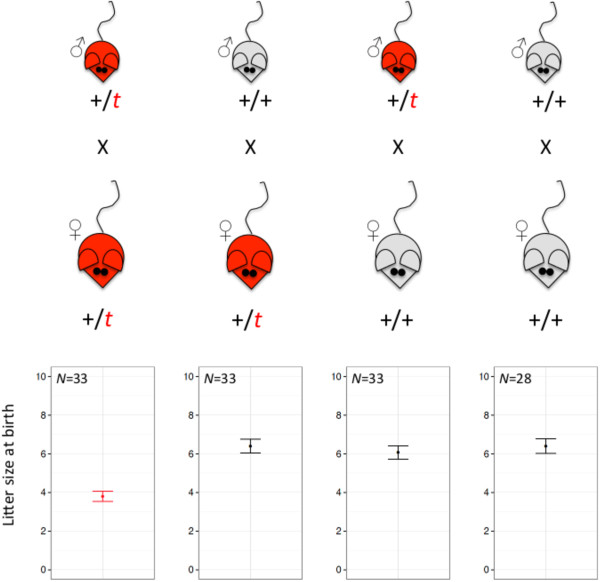
**Litter size at birth for four different mating crosses.** Litter sizes at birth for all four different mating crosses between +/*t* and +/+ mice are displayed. Plotted are back transformed model estimates [means] (glm) and the standard error of the mean.

Under standardised laboratory conditions, we analysed litter sizes at birth from four different mating crosses of +/*t* and +/+ house mice, originating from a wild population. A significant litter size reduction was observed when +/*t* females were mated with +/*t* males (*F*_3,123_=86.79, p-value<0.001) [37]. Model estimates of the mean are displayed in Figure 1. The litter size at birth of +/*t* females mated with +/*t* males was approximately 40% smaller than the litter size of any other mating cross (figure one, [37]).

Like all methods, this genetic tool comes with some limitations. Manipulation is only possible in one direction. Litter size can only be reduced, but not increased. Increasing litter size requires another method. Currently, litter size can be increased prepartum by inducing superovulation with gonadotrophins (house mice [38], sheep [39], bank voles [40]).

Furthermore, based on the mating design required for reduced litter sizes at birth, females with small and standard litters will either differ in their genotype (+/*t* or +/+) or the genotype (+/*t* or +/+) of the sire of their litter, or both. It is therefore not possible to completely disentangle other effects of the *t* besides the reduction in litter size. The *t* is known to affect functional sperm in males and behavioural studies have revealed that +/*t* females prefer +/+ males over +/*t* males, probably to avoid a reduction in litter size [37,41]. In the population from which our experimental animals derived, the *t* is associated with a unique MHC haplotype, and could thus play a role in *t* dependent mate choice [37]. Nevertheless, we do not expect mate choice to be a confounding factor in the setting presented here. First, litter size manipulation experiments are typically conducted in the laboratory and females are paired monogamously with males. In our laboratory crossings, both +/+ and +/*t* females did not differ in their propensity to conceive and to give birth when mated with +/*t* compared to +/+ males [37], indicating that they did not discriminate against +/*t* males. Second, the majority of experiments using litter size manipulations in house mice focus on the behaviour of the dams and/or the offspring, and there are up to now no indications that the *t* directly influences maternal behaviour.

A rather simple experimental design could thus help to answer to what extent prepartum versus postpartum number of offspring influences female reproductive costs, physiology and behaviour, by combining the genetic method to manipulate litter size prenatally with manipulations of litter size at birth. Furthermore, +/*t* females can alternatively be paired with a +/*t* male and with a +/+ male (full-sibs if required), or vice versa, therefore making it possible to compare data from the same female, once with a reduced and then with a standard litter size. In addition litter size reductions could help to reduce the number of mice born during experiments (in line with the 3R recommendations [42]).

## Conclusions

Experimental litter or clutch size manipulations are an important tool for gaining insight into the optimal litter size, and ultimately to understand life history strategies. Such manipulations can however cause substantial problems whenever the energy invested into the production of eggs or into gestation is ignored. Using a recessive lethal gene can help to reduce litters or clutches in a predictive way without interference after birth or oviposition. Recessive lethals can only generate litters that are reduced on average by 25%, but the transmission ratio distortion caused by the *t* haplotype in male house mice results in a litter size reduction of up to 50%. The earlier the recessive lethal property of the gene works, the better it controls for the prenatal costs of reproduction and potential prenatal adjustments to the litter size. Selfish genetic elements are assumed to be wide spread and often associated with recessive lethals, therefore similar methods could apply for a whole array of species [43]. This novel method allows the generation of smaller litters in a mammalian species without interfering after birth or using invasive techniques.

## Materials and methods

The data presented in this study were collected as part of a larger data set [37]. Data from experiment 1 and 2 of [37] were pooled for this analysis. In short, mice used were F1 to F3 descendants from wild house mice caught between 2006 and 2008 at a study population in Illnau, near Zurich, Switzerland. For more details on the free living study population see [37,44]. Experiments were conducted in an animal facility at the University of Zurich. Animal experimentation was approved by the Swiss Animal Experimentation Commission (Kantonale Tierversuchskommission, licence no. 97/2009). Prior to the experiments mice were kept in same-sex sibling groups after they had been removed from their parental cage at an age of 28 days. At that point a tissue sample was taken from each mouse for genotyping. The *t* haplotype was identified by scoring the genotype at the *Hba-ps4* locus [33] (for a detailed method see [37]). Mice used in the experiment inherited the *t* from the paternal, or maternal side. For simplicity, we always refer with +/*t* to heterozygous individuals, irrespective of whether they inherited the *t* from their mother or father. To our knowledge there are no imprinting effects known for the *t* haplotype.

During the experiments a male and a virgin female were kept together in a Macrolon type III cage (425 mm × 266 mm × 155 mm). The male was removed from the cage after 14 days and from day 19 onwards, cages were checked daily for new litters. After birth cages were searched for living and dead pups. All possible combinations of crosses between +/*t* and +/+ mice were used. In total 127 mating crosses were analysed. The exact numbers of each combination are indicated in Figure 1.

### Statistical analysis

All analyses were performed with R 2.15.1 [45]. A generalised linear model (glm) was used to test for an effect of the four different mating crosses on the litter size. The glm was fitted using a quasipoisson error distribution with a log-link function. Significance was tested by conducting F-tests, alpha was taken to be 0.05.

## Competing interests

The authors declare that they have no competing interests.

## Authors’ contributions

MF confirmed the method in yet unpublished work and wrote the main text. AL conceived the methodological idea, performed the mating crosses and contributed to the text. BK supervised the work and contributed to the text. All authors read and approved the final manuscript.
